# Restless leg syndrome and risk of all-cause dementia: a nationwide retrospective cohort study

**DOI:** 10.1186/s13195-023-01191-z

**Published:** 2023-03-06

**Authors:** Keun You Kim, Eun Hwa Kim, Myeongjee Lee, Junghee Ha, Inkyung Jung, Eosu Kim

**Affiliations:** 1 Department of Psychiatry, Institute of Behavioral Science in Medicine, Yonsei University College of Medicine, 50-1 Yonsei-ro, Seodaemun-gu, 03722 Seoul, Republic of Korea; 2 Department of Neuropsychiatry, Seoul Metropolitan Government-Seoul National University Boramae Medical Center, 20 Boramae-ro 5-gil, Dongjak-gu, 07061 Seoul, Republic of Korea; 3 Department of Biomedical Systems Informatics, Biostatistics Collaboration Unit, Yonsei University College of Medicine, 50-1 Yonsei-ro, Seodaemun-gu, 03722 Seoul, Republic of Korea; 4 Division of Biostatistics, Department of Biomedical Systems Informatics, Yonsei University College of Medicine, 50-1 Yonsei-ro, Seodaemun-gu, Seoul, 03722 Republic of Korea; 5 Brain Korea 21 FOUR Project for Medical Science, Yonsei University College of Medicine, 50-1 Yonsei-ro, Seodaemun-gu, Seoul, 03722 Republic of Korea

**Keywords:** Restless leg syndrome, Dementia, Alzheimer’s disease, Vascular dementia, Cohort study

## Abstract

**Background:**

Restless leg syndrome (RLS) is associated with poor sleep quality, depression or anxiety, poor dietary patterns, microvasculopathy, and hypoxia, all of which are known risk factors for dementia. However, the relationship between RLS and incident dementia remains unclear. This retrospective cohort study aimed to explore the possibility that RLS could be deemed as a non-cognitive prodromal feature of dementia.

**Methods:**

This was a retrospective cohort study using the Korean National Health Insurance Service-Elderly Cohort (aged ≥ 60). The subjects were observed for 12 years, from 2002 to 2013. Identifying patients with RLS and dementia was based on the 10th revised code of the International Classification of Diseases (ICD-10). We compared the risk of all-cause dementia, Alzheimer’s disease (AD), and vascular dementia (VaD) in 2501 subjects with newly diagnosed RLS and 9977 matched controls based on age, sex, and index date. The association between RLS and the risk of dementia was assessed using Cox regression hazard regression models. The effect of dopamine agonists on the risk of dementia among RLS patients was also explored.

**Results:**

The baseline mean age was 73.4, and the subjects were predominantly females (63.4%). The incidence of all-cause dementia was higher in the RLS group than that in the control group (10.4% vs 6.2%). A baseline diagnosis of RLS was associated with an increased risk of incident all-cause dementia (adjusted hazard ratio [aHR] 1.46, 95% confidence interval [CI] 1.24–1.72). The risk of developing VaD (aHR 1.81, 95% CI 1.30–2.53) was higher than that of AD (aHR 1.38, 95% CI 1.11–1.72). The use of dopamine agonists was not associated with the risk of subsequent dementia among patients with RLS (aHR 1.00, 95% CI 0.76–1.32).

**Conclusions:**

This retrospective cohort study suggests that RLS is associated with an increased risk of incident all-cause dementia in older adults, providing some evidence that requires confirmation through prospective studies in the future. Awareness of cognitive decline in patients with RLS may have clinical implications for the early detection of dementia.

## Background

Given the increase in the incidence and societal cost of dementia [1], identifying the risk of dementia in the preclinical stage is essential in terms of prevention. As well as an early cognitive sign, non-cognitive clinical signs can be associated with an increased risk of developing dementia. For instance, neuropsychiatric symptoms, such as depression, anxiety, and psychosis, can be presented at the preclinical stage of dementia, yielding the concept of mild behavioral impairment corresponding to mild cognitive impairment [2]. Sleep disorders, including primary insomnia, sleep-related breathing disorder, and rapid eye movement sleep behavior disorder, are also predictive signs of dementia [3]. Moreover, hearing loss is a strong risk factor for dementia [4]. These non-cognitive behavioral or sensorimotor signs have been of increased interest as potential early signs prior to overt dementia.

Restless leg syndrome (RLS), characterized by an urge to move the lower limbs and worsening symptoms at rest or in the evening [5], has multiple etiologies which may also be related to dementia. Previous studies reported that individuals with RLS tend to have sleep disturbance, depression or anxiety, poor dietary patterns, or obesity [6–8], all of which are also well-known risk factors or prodromal symptoms of overt dementia [3, 4]. RLS is also closely related to hypertension, heart disease, and stroke, suggesting vasculopathy in RLS [9, 10]. Neuroimaging and postmortem studies found that patients with RLS showed chronic and silent cerebral microvascular injury and gliosis, all of which are the risk factors for Alzheimer’s disease (AD) and vascular dementia (VaD) [11, 12]. Furthermore, the link between RLS and Parkinson’s disease [13–15] is possibly due to the shared pathophysiology of central dopamine dysfunction [15], which can also be found in AD [16, 17]. Iron deficiency and related hypoxia, another possible pathophysiology of RLS [14, 18], could induce cognitive impairment and dementia [19, 20].

Despite this potential link between RLS and risk factors for dementia, the relationship between RLS and dementia is yet to be elucidated. A few cross-sectional studies of whether patients with RLS tend to have cognitive deficits have presented conflicting results [21–25]. These studies have methodological limitations, including small sample sizes and a lack of temporal association. Hence, we aimed to explore the longitudinal association between RLS and incident all-cause dementia, including AD and VaD, using a nationwide retrospective large-sample cohort.

## Methods

### Study design and data source

National Health Insurance Service (NHIS) is a single mandatory public health insurance system in South Korea. NHIS covers approximately 97% of the South Korean population, while the remaining 3% who cannot afford to pay for NHIS are covered by the Medical Aid Program operated by the Korean government. This retrospective cohort study used data from the National Health Insurance Service-Elderly Cohort (NHIS-EC), a nationwide population-based cohort of subjects aged ≥ 60 from 2002, covering 12 years until 2013. The NHIS-EC randomly selected approximately 10% of the entire elderly population from both NHIS and Medical Aid Program (5.5 million) in South Korea and has a sample size of 558,147 [26]. The NHIS-EC database contains various information such as sociodemographic condition, insurance status, diagnosis (as defined by the 10th revised code of the International Classification of Diseases [ICD-10]), medical services (treatment and procedure), and costs claimed by hospitals.

This study was approved by the Institutional Review Board of Yonsei University Health System (4–2021-1051). The need for informed consent was waived owing to the study’s retrospective nature.

### Population selection

**Fig. 1:**
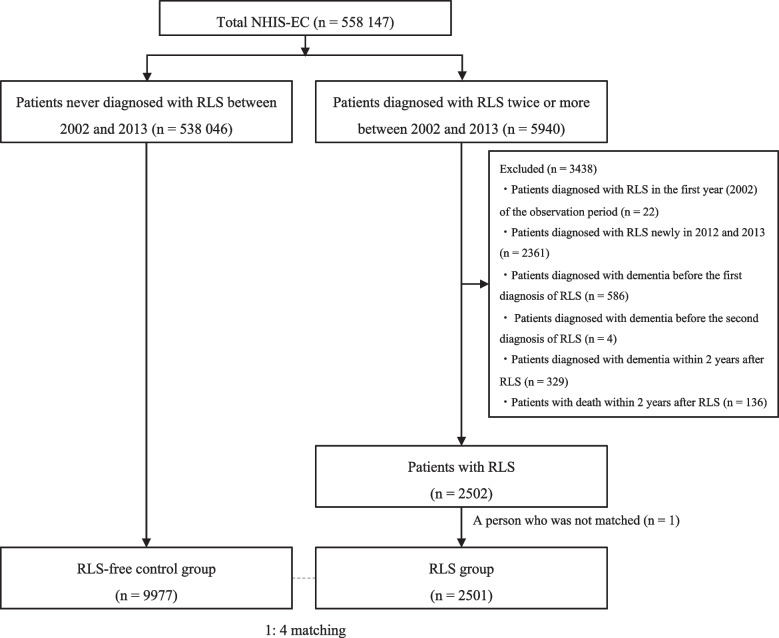
Flowchart of study population selection *Abbreviations*: NHIS-EC, National Health Insurance Service-Elderly Cohort; RLS, restless leg syndrome

The population selection flowchart is presented in Fig. 1. Since NHIS-EC was an administrative claim data, we could not apply formal diagnostic criteria of the International RLS Study Group [5] to identify patients with RLS. Instead, the presence of RLS was defined using the ICD-10 code G25.8. For diagnostic accuracy, patients with RLS were defined as those who had been diagnosed at least twice with this code (*n* = 5940). Meanwhile, RLS-free controls were defined as those who were never diagnosed with this code (*n* = 538,046). Among the 5940 patients with RLS, we excluded those diagnosed with dementia before the first diagnosis of RLS (*n* = 586) and the second diagnosis of RLS (*n* = 4). Considering the gradual onset of dementia, the minimal gap between the onset of RLS and any type of dementia was set at 2 years to minimize detection bias. Therefore, patients with RLS who were first diagnosed between 2012 and 2013 (*n* = 2361) or diagnosed with dementia within 2 years after RLS diagnosis (*n* = 329) were excluded. Additionally, patients with RLS in 2002, the first observation year of the NHIS-EC, were excluded due to the possibility that their first diagnosis was made before the observation period (*n* = 22). The patients were matched to controls in a maximum 1:4 ratio based on age, sex, and index date. Finally, 2501 patients with RLS and 9977 matched controls were included in the analysis.

All-cause dementia was defined as AD (ICD-10 F00 or G30), VaD (ICD-10 F01), and other types of dementia (ICD-10 F02, F03, F10.7, G23.1, G31.0, G31.1, G31.2, or G31.8). Dementia patients were defined as those diagnosed at least twice with the relevant ICD code(s) to minimize the possibility of over-classification of cases due to using ICD codes instead of formal diagnostic criteria. If patients had ICD codes of both AD and VaD, we classified these patients into VaD as a primary diagnosis following a previous study using the same database [27]. Among all-cause dementia cases (*n* = 874), 54.4% (*n* = 475) was AD, and 22.2% (*n* = 194) was VaD, which was consistent with the general epidemiology of dementia in South Korea [28]. Patients with other types of dementia were not analyzed separately because of heterogeneous disease entities and small sample sizes.

### Comorbidity

To adjust for comorbidities (e.g., cardiac disease, cerebrovascular disease, renal failure, liver diseases, malignancies, and diabetes mellitus), Quan’s algorithm of Charlson Comorbidity Index (CCI) [29] was used, which is known to predict mortality adequately [30]. Given that the original CCI calculation includes dementia diagnosis, we calculated CCI except for dementia because it was our primary outcome variable. In addition to CCI, a history of schizophrenia, mood disorders (depression and bipolar disorder), anxiety disorders, Parkinson’s disease, iron deficiency anemia, and sleep disorders (insomnia, hypersomnia, sleep-related breathing disorder, narcolepsy, sleepwalking, sleep terror, and nightmare) were considered as covariates.

### Statistical analysis

Chi-square for categorical variables and Mann–Whitney *U* test for continuous variables due to non-normality were used to compare the baseline characteristics between patients with RLS and RLS-free controls. Cox proportional hazards regression models were applied to explore the association between RLS and the risk of dementia after adjusting for age, sex, income, residence, CCI, and history of other comorbidities. Among the Cox regression models, we used the Fine–Gray subdistribution hazard model with mortality as a competing risk given the old age of the study population. The proportional hazard assumption was satisfied in our Cox model (Schoenfeld individual test *p*-value > 0.05).

Sensitivity analyses were performed using four different models. In model 1, dementia was defined as the prescription of anti-dementia medications (donepezil, rivastigmine, galantamine, and memantine) at least twice and a diagnosis of the ICD-code of dementia. Although these medications were approved for only AD (rivastigmine additionally for Parkinson’s disease dementia), they can be used for cognitive symptoms in other types of dementia based on recommendations from multiple guidelines [31–33]. The previous study revealed that the definition of all-cause dementia by ICD-10 code plus anti-dementia medications had a positive predictive value of 94.7% when reviewing the medical records of 972 patients in two hospitals [34]. In model 2, medication history was added to the ICD code to define RLS. Patients with RLS ICD-code (G25.8) who had taken dopamine agonists (ropinirole or pramipexole) twice or more were regarded as patients with RLS (*n* = 1458). In this sensitivity model, we excluded patients with Parkinson’s disease because they could also take dopamine agonists. In model 3, patients taking antipsychotic agents were excluded because the antidopaminergic property of antipsychotic agents could lead to a misdiagnosis of RLS (*n* = 2482). The following antipsychotic agents approved in South Korea were used in this study: haloperidol, sulpiride, chlorpromazine, perphenazine, pimozide, risperidone, olanzapine, quetiapine, paliperidone, amisulpride, aripiprazole, ziprasidone, clozapine, blonanserin, and zotepine. In model 4, patients with RLS only diagnosed by psychiatrists or neurologists were included (*n* = 1154) to preclude the possible misdiagnosis by non-expert physicians.

To evaluate the effect of dopamine agonists (pramipexole and ropinirole) on the development of dementia, the risk of dementia was compared after dividing RLS patients by dopamine agonist use. Patients with RLS who were prescribed pramipexole or ropinirole at least once were considered dopamine agonist users. All missing data were addressed using listwise deletion. Data processing and statistical analyses were performed using SAS version 9.4 (SAS Institute, Cary, NC, USA). Statistical significance was set at a two-tailed *p*-value of < 0.05.

## Results

### Characteristics of the study population

The mean age was 73.4 years, and the subjects were predominantly females (65.1%). Among the 12,478 subjects, 874 (7.0%) subjects developed all-cause dementia (475 [54.4%] subjects, AD; 194 [22.2%] subjects, VaD). The characteristics of the study population are described in Table 1. Compared with the control group, the RLS group had a higher chance of having sleep, mood, and anxiety disorders and Parkinson’s disease and had higher overall CCI scores. The proportions of low-income subjects and those living in rural areas were comparable between the two groups. The incidence of all-cause dementia was significantly higher in the RLS group than in the control group (10.4% vs. 6.2%, *p* < 0.001). Likewise, the incidence rates of AD and VaD (5.6% and 2.6%, respectively) were also higher in the RLS group than in the control group (3.4% and 1.3%, respectively).

**Table 1:** Characteristics of the study population

	Control group (*n* = 9977)	RLS group (*n* = 2501)	*p*-value^a^
Age at enrollment (years)	73.35 ± 5.18	73.40 ± 5.25	0.839
Sex			0.936
Male	3474 (34.8)	873 (34.9)	
Female	6503 (65.2)	1628 (65.1)	
Income level by insurance fee			0.213
Bottom 0 to 20th percentile	2189 (21.9)	520 (20.8)	
Bottom 20th to 100th percentile	7788 (78.1)	1981 (79.2)	
Region of residence			0.368
Urban	4092 (41.0)	1001 (40.0)	
Rural	5885 (59.0)	1500 (60.0)	
CCI score	1.22 ± 1.41	1.91 ± 1.75	< 0.001
Comorbidities			
Schizophrenia or other psychotic disorder	19 (0.2)	20 (0.8)	< 0.001
Mood disorder	434 (4.4)	368 (14.7)	< 0.001
Anxiety disorder	636 (6.4)	412 (16.5)	< 0.001
Parkinson’s disease	38 (0.4)	115 (4.6)	< 0.001
Iron deficiency anemia	165 (1.7)	71 (2.8)	< 0.001
Sleep disorder^b^	361 (3.6)	316 (12.6)	< 0.001
Incidence of dementia			
All-cause dementia	614 (6.2)	260 (10.4)	< 0.001
AD	336 (3.4)	139 (5.6)	< 0.001
VaD	130 (1.3)	64 (2.6)	< 0.001
All-cause mortality	490 (4.9)	120 (4.8)	0.814
Follow-up duration (years)	3.94 ± 1.62	3.88 ± 1.60	0.068

Values are expressed as the mean ± standard deviation or *n* (%)

*Abbreviations*: AD, Alzheimer’s disease; CCI, Charlson Comorbidity Index; RLS, restless leg syndrome; VaD, Vascular dementia

^a^Continuous variables by Mann–Whitney *U* test and categorical variables by the chi-square test

^b^Insomnia, hypersomnia, sleep-related breathing disorder, narcolepsy, sleepwalking, sleep terror, and nightmare

### Risk of dementia in patients with RLS

**Fig. 2:**
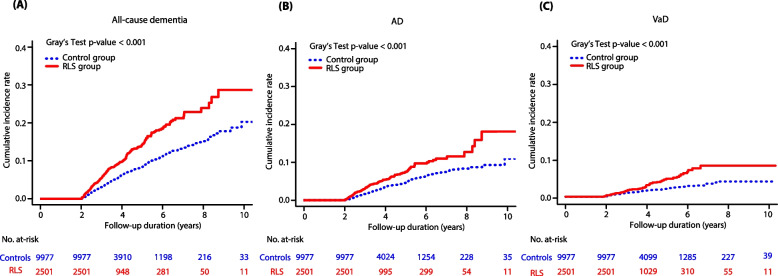
Cumulative incidence of the risk of dementia in patients with RLS and controls *Abbreviations*: AD, Alzheimer’s disease; RLS, restless leg syndrome; VaD, vascular dementia

Stratified Cox regression analysis (Table 2) showed that the risk of all-cause dementia was 1.74 times higher in the RLS group than in the control group (95% confidence interval [CI] 1.51–2.02, *p* < 0.001). This significance remained after adjustment for covariates (adjusted hazard ratio [aHR] 1.46, 95% CI 1.24–1.72, *p* < 0.001). Furthermore, the presence of RLS was significantly associated with an increased risk of both AD (aHR 1.38, 95% CI 1.11–1.72) and VaD (aHR 1.81, 95% CI 1.30–2.53) (Table 2). The cumulative longitudinal influence of RLS on dementia risk is shown in Fig. 2. The Kaplan–Meier survival curve with Gray’s test revealed that the RLS group had a higher incidence of all-cause dementia, AD, and VaD than the control group during the observation period (all *p* < 0.001, Fig. 2A–C). Notably, the cumulative incidence rate was 0 in the first 2 years, which we set as the minimum duration between incident RLS and subsequent dementia, considering the probable insidious influence of RLS on the brain.

**Table 2:** Risk of dementia between the RLS and control groups

	Cases, *n* (%)	Stratified Cox regression model			
		Crude HR (95% CI)	*p*-value	aHR^a^ (95% CI)	*p*-value
**All-cause dementia**					
RLS group	260 (10.4)	1.74 (1.51–2.02)	< 0.001	1.46 (1.24–1.72)	< 0.001
Control group	614 (6.2)	1 (reference)		1 (reference)	
**AD**					
RLS group	139 (5.6)	1.68 (1.38–2.05)	< 0.001	1.38 (1.1–1.72)	0.004
Control group	336 (3.4)	1 (reference)		1 (reference)	
**VaD**					
RLS group	64 (2.6)	1.98 (1.46–2.69)	< 0.001	1.81 (1.30–2.53)	< 0.001
Control group	130 (1.3)	1 (reference)		1 (reference)	

*Abbreviations*: AD, Alzheimer’s disease; aHR, adjusted hazard ratio; CCI, Charlson Comorbidity Index; CI, confidence interval; HR, hazard ratio; RLS, restless leg syndrome; VaD, Vascular dementia

^a^Adjusted for income, region, CCI score, history of sleep disorder, schizophrenia or other psychotic disorders, mood disorder, anxiety disorder, Parkinson’s disease, and iron deficiency anemia

### Sensitivity analysis

Table 3 shows the sensitivity analyses in the four different models. In model 1 (Table 3, the leftmost panel), Cox regression analyses revealed a significantly higher risk of all-cause dementia (aHR 1.51, 95% CI 1.22–1.88, *p* < 0.001) and VaD (aHR 2.238, 95% CI 1.58–3.43, *p* < 0.001) in the RLS group. For AD, a non-significant but higher risk trend was observed (aHR 1.23, 95% CI 0.94–1.62, *p* = 0.133). In model 2 (Table 3, second panel from the left), the risks of all-cause dementia (aHR 1.40, 95% CI 1.21–1.76, *p* = 0.004) and VaD (aHR 1.62, 95% CI 1.01–2.58, *p* = 0.045) were significantly higher in the RLS group than in the control group. The risk of AD was also higher in the RLS group, with a marginal significance (*p* = 0.055). In model 3 (Table 3, third panel from the left), the risks of all-cause dementia (aHR 1.44, 95% CI 1.22–1.70, *p* < 0.001), AD (aHR 1.37, 95% CI 1.10–1.71, *p* = 0.006), and VaD (aHR 1.79, 95% CI 1.28–2.50, *p* = 0.001) remained higher in the RLS group than in the control group. Similarly, in model 4 (Table 3, the rightmost panel), the RLS group showed a significantly increased risk of all-cause dementia (aHR 1.86, 95% CI 1.52–2.31, *p* < 0.001), AD (aHR 1.76, 95% CI 1.32–2.34, *p* < 0.001), and VaD (aHR 2.72, 95% CI 1.76–4.20, *p* < 0.001).

**Table 3:** Sensitivity analyses of risk for dementia in the RLS and control groups

	**Model 1: Definition of dementia by ICD-10 codes plus anti-dementia medications**^b^			**Model 2: Definition of RLS by ICD-10 code plus dopamine agonists**^c^			**Model 3: Exclusion of patients taking antipsychotic agents**^d^			**Model 4: Diagnosis of RLS by psychiatrists or neurologists**		
	Cases, *n* (%)	aHR^a^ (95% CI)	*p*-value	Cases, *n* (%)	aHR^a^ (95% CI)	*p*-value	Cases, *n* (%)	aHR^a^ (95% CI)	*p*-value	Cases, *n* (%)	aHR^a^ (95% CI)	*p*-value
**All-cause dementia**												
RLS group	147 (6.2)	1.51 (1.22–1.88)	< 0.001	129 (8.8)	1.40 (1.12–1.76)	0.004	256 (10.3)	1.44 (1.22–1.70)	< 0.001	152 (13.2)	1.86 (1.50–2.31)	< 0.001
Control group	332 (3.4)	1 (reference)		316 (5.5)	1 (reference)		611 (6.2)	1 (reference)		316 (6.9)	1 (reference)	
**AD**												
RLS group	94 (3.9)	1.23 (0.94–1.62)	0.133	66 (4.5)	1.36 (0.94–1.85)	0.055	137 (5.5)	1.37 (1.10–1.71)	0.006	87 (7.5)	1.76 (1.32–2.34)	< 0.001
Control group	236 (2.4)	1 (reference)		162 (2.8)	1 (reference)		334 (3.4)	1 (reference)		176 (3.8)	1 (reference)	
**VaD**												
RLS group	45 (1.9)	2.33 (1.58–3.43)	< 0.001	30 (2.1)	1.62 (1.01–2.58)	0.045	63 (2.5)	1.79 (1.28–2.50)	0.001	37 (3.2)	2.72 (1.76–4.20)	< 0.001
Control group	78 (0.8)	1 (reference)		68 (1.2)	1 (reference)		130 (1.3)	1 (reference)		58 (1.3)	1 (reference)	

*Abbreviations*: AD, Alzheimer’s disease; aHR, adjusted hazard ratio; CCI, Charlson Comorbidity Index; CI, confidence interval; ICD, International Classification of Diseases; RLS, restless leg syndrome; VaD, Vascular dementia

^a^Adjusted for income, region, CCI score, history of sleep disorder, schizophrenia or other psychotic disorders, mood disorder, anxiety disorder, Parkinson’s disease, and iron deficiency anemia

^b^Anti-dementia medications include donepezil, rivastigmine, galantamine, and memantine

^c^Patients with Parkinson’s disease were excluded from these analyses. Dopamine agonists include pramipexole and ropinirole

^d^Antipsychotic agents include haloperidol, sulpiride, chlorpromazine, perphenazine, pimozide, risperidone, olanzapine, quetiapine, paliperidone, amisulpride, aripiprazole, ziprasidone, clozapine, blonanserin, and zotepine

### Effect of dopamine agonists on the relationship between RLS and dementia

In the RLS group, 1942 (77.65%) had used dopamine agonists (e.g., pramipexole or ropinirole). Stratified Cox regression analysis showed no significant difference in the risk of all-cause dementia between dopamine agonist users and non-users (Table 4).

**Table 4:** Effect of dopamine agonist on the development of dementia in patients with RLS

	All-cause dementia cases (*n* (%))	Crude HR (95% CI)	*p*-value	aHR^a^ (95% CI)	*p*-value
Patients with RLS with dopamine agonist use^b^	190 (9.8)	0.97 (0.74–1.28)	0.845	1.00 (0.76–1.32)	0.983
Patients with RLS without dopamine agonist use^b^	70 (12.5)	1 (reference)		1 (reference)	

*Abbreviations*: aHR, adjusted hazard ratio; CI, confidence interval; HR, hazard ratio; RLS, restless leg syndrome

^a^Adjusted for age, sex, income, region, CCI score, history of sleep disorder, schizophrenia or other psychotic disorders, mood disorder, anxiety disorder, Parkinson’s disease, and iron deficiency anemia

^b^Dopamine agonists are pramipexole and ropinirole

## Discussion

This nationwide population-based retrospective cohort study found that patients with RLS had an increased risk of incident dementia. Sensitivity analyses in four models adjusted for different factors presented the same results, except for non-significance in the risk of AD in two of the four models. Our findings suggest the possibility of RLS as a risk factor or a prodromal sign of dementia.

Previous cross-sectional studies of cognitive function and RLS have shown conflicting results. On the one hand, some studies reported that patients with RLS presented deficits in attention and frontal lobe function tests, not memory or global cognition [21–23]. Difficulties in attention or frontal lobe tasks may be attributed to sleep deprivation [21, 22] or depression [23]. In contrast, some studies demonstrated that patients with RLS did not show impaired cognition [24, 25] and even showed partially better performance than sleep-deprived controls did due to adaptation to sleep loss [25]. Our longitudinal study of a large sample cohort suggests the possibility that RLS may precede the deterioration in global cognition leading to dementia.

The underlying mechanism of the possible link between RLS and subsequent dementia is unclear. Poor sleep quality, depression or anxiety, lack of a Mediterranean diet, and obesity are risk factors for RLS [6–8]. These poor quality-of-life indicators are also associated with an increased risk of dementia [3, 4]. RLS is also associated with poor cardiovascular conditions and stroke [9, 10], which is responsible for the development of dementia. Postmortem and neuroimaging studies reported that patients with RLS had silent and chronic microvascular disease and gliosis in the brain [11, 12]. Moreover, prior studies that patients with RLS had decreased gray and white matter volume [35, 36] support the possible link between RLS and central neurodegeneration.

RLS symptoms inevitably cause sleep disturbance, especially sleep induction, which could be associated with an increased risk of dementia. A meta-analysis reported that insomnia increases the risk of dementia by 27%, with a sleep duration of approximately 6 h for a lower risk of dementia [3]. Reduced sleep provokes β-amyloid accumulation via reduced slow-wave sleep [37]. Although we adjusted for sleep disorders, including primary insomnia and sleep-related breathing disorders, it is still uncertain whether our study results of a significant association between RLS and subsequent dementia were due to sleep disturbance.

Central dopamine and iron deficit are other possible pathophysiologies shared by RLS and dementia. Decreased dopamine receptor was reported not only in RLS and Parkinson’s disease but also in AD and VaD [17, 38]. In contrast, a dopaminergic tone would increase in AD patients with psychotic symptoms (e.g., hallucinations and delusions) [39]. Likewise, iron deficiency anemia, one of the core pathologies of RLS, could induce cognitive impairment or dementia via the hypoxia pathway [19]. Meanwhile, recent studies demonstrated that high hemoglobin and brain iron deposition were also associated with a high risk of AD [40, 41], possibly due to iron-induced β-amyloid accumulation [41]. Our result of higher HR in VaD than AD might imply these bidirectional influences of dopamine and iron in the brain. The possibility of more vasculopathy than AD pathology in RLS is also supported by the postmortem study in which patients with RLS presented microvascular disease without β-amyloid aggregation [11]. However, since our study is an observational retrospective cohort study, causal inference between RLS and dementia cannot be confirmed. Considering that neuropathologic change begins two decades before the diagnosis of dementia [42], approximately 4 years of follow-up in our study might not be a sufficient duration for such an inference. Further replicative longitudinal prospective studies regarding cardiovascular/metabolic diseases and lifestyle indicators are needed to explore their relationship, especially based on neuroimaging, cognitive function tests, and expert-based assessment.

In this study, dopamine agonists did not affect the cumulative incidence of dementia in the RLS group. Dopamine transmission is known to be reduced in AD, and dopamine agonists could potentially improve cortical plasticity [17]. Considering that this study was not a randomized controlled study, patients with RLS who use dopamine agonists might have worse symptoms than non-users, which could have masked the effect of the medication. Furthermore, we could not assess the influence of other medications, such as oral iron, benzodiazepines, or α2δ ligands [14], because of their non-approval in Korea. Future prospective well-controlled studies will help determine which medications attenuate the development of dementia in patients with RLS.

To the best of our knowledge, this is the first study to evaluate whether incident RLS is associated with increased dementia risk. Using a nationwide elderly cohort with a large sample size, we revealed a possible temporal relationship between RLS and dementia through a follow-up of a maximum of 10 years. Given the insidious development of dementia, a gap of 2 years between RLS and dementia was set to minimize detection bias. Furthermore, an elderly cohort aged ≥ 60 was selected to capture the substantial population of dementia, with adjustments for not only comorbidities but also socioeconomic status.

The major limitation of this study is that, although the administrative database used in this research has been widely used, identifying patients with RLS and dementia by ICD-10 codes (not formal diagnostic criteria) might have led to a few types of biases. First, patients who mimicked RLS and dementia might have been included (misclassification bias). However, RLS is prone to underdiagnosis, especially in Asian countries, and the prevalence of RLS in our study is lower than that in other epidemiologic studies [43], suggesting that the selection of patients with RLS in this study had high sensitivity. The sensitivity analysis model 4 with the diagnosis of RLS by experts (psychiatrists or neurologists) also showed a significant association between RLS and an increased risk of dementia. Second, applying our strict criteria defining patients with RLS or dementia (at least twice diagnoses of ICD-10 code) might provoke additional selection bias. Subjects with multiple visits might have had severer manifestations or been more accessible to health care services. Third, classifying AD and VaD from cases of all-cause dementia might be inaccurate considering the ICD code-based diagnosis, although the proportion of AD and VaD in our study was consistent with general epidemiology [28]. Fourth, identifying all-cause dementia by cholinesterase inhibitors (donepezil, rivastigmine, galantamine) and memantine in the sensitivity analysis model 1 might have excluded patients with dementia other than AD. These medications are approved for AD despite the evidence supporting their usage for other types of dementia [31–33] and previously reported high positive predictive value of this method for all-cause dementia [34]. A prospective study using formal diagnostic criteria, brain imaging, and cognitive testing is needed to confirm the relationship between RLS and dementia.

This study also has some other limitations. First, as previously mentioned, this observational epidemiological study cannot confirm the causal relationship. Also, approximately 4 years of the mean follow-up duration might not be enough to confirm the (causal) relationship between RLS and dementia. Second, the results are possibly confounded by other hidden factors, despite adjustment for covariates. Especially, a lack of a Mediterranean diet or poor dietary intake, associated with both RLS [6] and dementia [4], was unavailable to be identified in this study. Moreover, the study population was predominantly female and aged ≥ 60, which might have led to a selection bias. Third, we could not evaluate other types of dementia, mainly Parkinson’s disease dementia or Lewy body dementia, due to the insufficient sample size. Finally, the severity of RLS, which would affect the outcome variables, was unavailable in our data. Further studies on the pathophysiological mechanism or protective factors between the two diseases may be valuable.

## Conclusions

We found that incident RLS is associated with an elevated risk of dementia in older adults, suggesting that RLS could be regarded as a newly identified risk factor or prodromal sign of dementia. If so, regular check-ups for cognitive decline in older patients with RLS may facilitate earlier detection and intervention for those with dementia risk. Prior to realizing this proposal, however, some evidence provided here will remain to be confirmed; more extensive and mechanistic studies should follow.

## Data Availability

Data used in this study are not available for public access. However, further information can be requested from the corresponding author at reasonable request.

## Abbreviations

AD: Alzheimer’s disease
aHR: Adjusted hazard ratio
CCI: Charlson Comorbidity Index
CI: Confidence interval
HR: Hazard ratio
ICD: International Classification of Diseases
NHIS-EC: National Health Insurance Service-Elderly Cohort
RLS: Restless leg syndrome
VaD: Vascular dementia
